# The Role of microRNA-210 in the Pathogenesis and Diagnosis of Preeclampsia—A Systematic Review

**DOI:** 10.3390/jcm14217593

**Published:** 2025-10-26

**Authors:** Oana Eliza Cretu, Alina Alexandra Dirlau, Adrian Valeriu Neacsu, Adina Elena Nenciu, Iuliana Ceausu

**Affiliations:** Obstetrics and Gynecology Department, Carol Davila University of Medicine and Pharmacy, 020021 Bucharest, Romania; oana-eliza.cretu@drd.umfcd.ro (O.E.C.); adrian-valeriu.neacsu@drd.umfcd.ro (A.V.N.); adina-elena.afloarea@drd.umfcd.ro (A.E.N.); iuliana.ceausu@umfcd.ro (I.C.)

**Keywords:** preeclampsia, microRNA-210, trophoblast dysfunction, mitochondrial metabolism, angiogenesis, hypoxia, HIF-1α, biomarker, RNA-based therapy, personalised obstetric care

## Abstract

**Background**: Preeclampsia is a complex hypertensive disorder of pregnancy associated with significant maternal and foetal morbidity and mortality. Its pathogenesis involves placental hypoxia, oxidative stress, and impaired trophoblast invasion. Recent evidence highlights the role of microRNAs, particularly microRNA-210 (miR-210), in the molecular disruptions underlying preeclampsia. Aim: This study aims to explore the pathogenic, diagnostic, and therapeutic significance of miR-210 in preeclampsia, with emphasis on its molecular mechanisms, biomarker potential, and prospects as a therapeutic target. **Methods**: A systematic narrative review was conducted following PRISMA guidelines. A total of 498,184 articles were identified through eight scientific databases, and, after duplicate removal and eligibility screening, 111 peer-reviewed studies published between 2015 and 2025 were included in the final analysis. The selected literature focused on miR-210’s expression in placental tissue and maternal circulation, its molecular targets, and its clinical relevance. **Results**: miR-210 is consistently upregulated in preeclamptic placentas and maternal plasma. It contributes to shallow trophoblast invasion, impaired angiogenesis, mitochondrial dysfunction, and the activation of a hypoxia-induced HIF-1α feedback loop. These mechanisms are central to the disease’s pathophysiology. Clinically, miR-210 demonstrates high stability in circulation and early detectability, making it a promising diagnostic and prognostic biomarker. Experimental models have also demonstrated the therapeutic potential of miR-210 inhibition using antisense oligonucleotides or HIF-1α modulators. **Conclusions**: miR-210 is both a marker and mediator of preeclampsia. Its integration into diagnostic protocols and therapeutic strategies, alongside clinical validation and standardisation, may enhance early detection and personalised care in high-risk pregnancies.

## 1. Introduction

Each year, approximately 3–6% of pregnancies worldwide are affected by preeclampsia (PE), a major contributor to maternal and foetal morbidity and mortality [1]. In both high- and low-resource settings, PE continues to pose serious health risks due to its unpredictable onset and progression [2]. Clinically, PE is characterised by new-onset hypertension after 20 weeks of gestation, frequently accompanied by proteinuria or evidence of maternal organ dysfunction [3,4].

Despite decades of research, the pathogenesis of PE remains incompletely understood. A growing body of evidence suggests that abnormal placentation and persistent hypoxia play a central role in the development of the disease [5].

In normal pregnancy, extravillous cytotrophoblasts invade the maternal decidua and remodel the spiral arteries to create a low-resistance, high-capacitance vascular system [6]. In PE, inadequate trophoblast invasion and poor vascular remodelling lead to placental ischemia, oxidative stress, and the release of anti-angiogenic factors such as soluble fms-like tyrosine kinase-1 (sFlt-1) and soluble endoglin (sEng) [7,8].

These factors suppress proangiogenic signalling pathways (VEGF, PlGF), contributing to systemic endothelial dysfunction and clinical manifestations of PE [9]. In recent years, epigenetic regulation, particularly via microRNAs (miRNAs), has emerged as a critical element in the molecular landscape of PE.

Among these, microRNA-210 (miR-210) has been consistently found to be upregulated in placental tissues and maternal plasma from women with PE [10,11]. miR-210 is known as a hypoxia-inducible miRNA, regulated primarily by HIF-1α (hypoxia-inducible factor 1-alpha), and acts as a master regulator of mitochondrial metabolism, angiogenesis, and trophoblast function under low-oxygen conditions [12].

The overexpression of miR-210 has been associated with impaired trophoblast invasion, mitochondrial dysfunction, and a pro-inflammatory placental environment—all key features in the pathophysiology of PE [13,14].

Additionally, circulating levels of miR-210 have shown potential as non-invasive biomarkers for the early detection of PE, although clinical validation is still ongoing [15]. This review aims to provide a comprehensive summary of the current knowledge regarding the role of miR-210 in the pathogenesis, diagnosis, and potential treatment strategies of preeclampsia. Furthermore, we explore the molecular mechanisms regulated by miR-210, including its downstream targets, and discuss the prospects of using miRNA-based diagnostics and therapeutics in PE management.

### 1.1. Biogenesis and Regulation of miR-210

microRNA-210 (miR-210) is one of the most thoroughly investigated hypoxia-inducible microRNAs and plays a critical role in cellular adaptation to low-oxygen conditions, including those seen in the pathological placental environment of preeclampsia. Its consistent upregulation in both the placental tissue and maternal circulation in women with preeclampsia has positioned it as a key molecular player in the disease process [16].

At the genomic level, miR-210 is transcribed from a non-coding region on chromosome 11p15.5, an area also associated with imprinting and epigenetic regulation. Transcription is carried out by RNA polymerase II, generating a primary transcript (pri-miR-210) that structurally resembles messenger RNA, possessing both a 5′ cap and a poly-A tail [17]. This pri-miRNA is cleaved in the nucleus by the Drosha-DGCR8 microprocessor complex into a ~70 nucleotide precursor known as pre-miR-210, which folds into a characteristic stem-loop structure. The precursor is then exported to the cytoplasm via Exportin-5, a Ran-GTP-dependent nuclear transport protein [18].

In the cytoplasm, the Dicer enzyme further processes the pre-miRNA into a ~22-nucleotide double-stranded RNA duplex. One strand, referred to as the guide strand, is incorporated into the RNA-induced silencing complex (RISC), where it guides the complex to complementary sequences in the 3′ untranslated regions (UTRs) of target messenger RNAs, leading to translational repression or degradation of the target transcripts [19].

The transcriptional regulation of miR-210 is tightly controlled by the oxygen tension in the cellular environment. Hypoxia-inducible factor 1-alpha (HIF-1α) is the principal regulator of miR-210 and plays a pivotal role in its activation under hypoxic conditions. In low-oxygen states, HIF-1α stabilises and translocates to the nucleus, where it binds to hypoxia response elements (HREs) in the promoter region of the MIR210 gene, driving transcription [20]. This mechanism has been confirmed in placental trophoblast models and human tissue using techniques such as chromatin immunoprecipitation (ChIP) and luciferase reporter assays [21].

In addition to hypoxia, other regulatory pathways also influence miR-210 expression. The NF-κB signalling cascade, often activated in pro-inflammatory environments, can contribute to miR-210 upregulation in certain cell types, hinting at a possible link between inflammation and microRNA dysregulation in preeclampsia [22]. Similarly, the tumour suppressor protein p53 has been implicated in the regulation of hypoxia-sensitive microRNAs including miR-210, particularly in response to oxidative stress [23]. Epigenetic mechanisms such as promoter methylation and histone modification can also modulate MIR210 transcription by altering chromatin accessibility under stress conditions commonly observed in preeclamptic placentas [24].

Once mature, miR-210 exerts its biological functions by targeting a broad array of mRNAs involved in mitochondrial metabolism, angiogenesis, DNA repair, and trophoblast differentiation. Among its best-characterised targets are ISCU1/2, which encode iron–sulphur cluster assembly proteins critical for mitochondrial electron transport. Repression of ISCU by miR-210 leads to impaired mitochondrial respiration, increased reactive oxygen species (ROS) production, and amplified oxidative stress [25]. Other notable targets include EFNA3, a negative regulator of angiogenesis, and genes such as HOXA9 and E2F3, which modulate trophoblast proliferation and invasion [26]. These downstream effects of miR-210 are directly relevant to the pathophysiological features of preeclampsia.

Reduced mitochondrial activity, impaired vascular remodelling, and shallow trophoblast invasion are all hallmarks of the disorder and are exacerbated by sustained miR-210 expression. A self-reinforcing feedback loop has also been proposed, wherein placental hypoxia induces HIF-1α, which elevates miR-210, which in turn suppresses mitochondrial function, leading to further ROS production and continued HIF-1α stabilisation.

This cycle contributes to placental damage and systemic endothelial dysfunction, accelerating disease progression [27]. From a clinical perspective, the detection of elevated miR-210 levels in maternal blood has sparked interest in its potential as a non-invasive biomarker for preeclampsia.

Its stability in plasma and serum, even under variable storage conditions, gives miR-210 a distinct advantage over protein biomarkers. Significant differences in circulating miR-210 levels have been reported between normotensive and preeclamptic pregnancies, including during the second trimester, suggesting its potential utility for early screening and risk stratification [28].

Meta-analyses indicate that miR-210 exhibits promising diagnostic performance, with pooled sensitivities and specificities above 80 per cent in some studies. However, large-scale prospective trials are still required to validate its clinical applicability across diverse populations and stages of disease [29].

### 1.2. Mechanistic Role of miR-210 in Trophoblast Biology

Trophoblast cells play a fundamental role in the establishment and maintenance of pregnancy by mediating implantation, maternal–foetal communication, and placental development. The invasive capacity of extravillous trophoblasts and their ability to remodel maternal spiral arteries are essential for adequate placental perfusion. Disruption of these processes is a hallmark of preeclampsia and recent studies have highlighted the role of miR-210 in this context.

Elevated levels of miR-210 in preeclamptic placentas have been shown to impair multiple aspects of trophoblast function including invasion, differentiation, mitochondrial metabolism, and response to hypoxic stress [30]. Under normal conditions, extravillous trophoblasts invade the decidua and modify maternal vasculature to establish a low-resistance, high-capacitance blood flow system.

In preeclampsia, this invasive potential is reduced and miR-210 has been implicated as a negative regulator of this capacity. miR-210 suppresses genes involved in extracellular matrix degradation and cell motility such as THSD7A and MNT, thereby limiting the trophoblast’s ability to penetrate the maternal endometrium [31]. Furthermore, miR-210 downregulates HSD17B1, a gene important for hormonal regulation and local oestrogen production, contributing to a microenvironment less conducive to normal trophoblast behaviour [32].

At the metabolic level, miR-210 directly targets key mitochondrial genes including ISCU1/2 and SDHD. The repression of these genes disrupts electron transport chain function, leading to decreased ATP production and increased accumulation of reactive oxygen species. This oxidative stress impairs trophoblast proliferation and promotes cellular senescence, contributing to the shallow invasion observed in preeclampsia [33].

The impaired mitochondrial function is particularly detrimental in trophoblasts due to their high energy demand during early placental development. In addition, miR-210 influences trophoblast survival and differentiation through modulation of the Notch and VEGF signalling pathways.

Downregulation of EFNA3 by miR-210 inhibits angiogenic signalling, which not only affects vascular development but also limits trophoblast–endothelial interactions critical for vessel remodelling [34].

Evidence also suggests that persistent overexpression of miR-210 promotes a shift toward a more glycolytic metabolic phenotype, which may serve as an adaptive mechanism under hypoxia but compromises long-term placental efficiency [35]. Collectively, these findings demonstrate that miR-210 acts as a molecular brake on normal trophoblast function. Its upregulation in preeclamptic placentas disrupts key cellular pathways and contributes directly to the placental pathology observed in the disorder [36].

### 1.3. miR-210 as a Biomarker in Preeclampsia

Early detection of preeclampsia remains a clinical priority due to the unpredictable nature of the disorder and its association with significant maternal and foetal morbidity. Traditional diagnostic markers such as blood pressure and proteinuria often reflect late-stage manifestations and lack the sensitivity required for early prediction. In this context, miR-210 has emerged as a promising biomarker owing to its consistent upregulation in preeclamptic pregnancies and its stability in maternal circulation [37]. Elevated levels of miR-210 have been reported in the plasma and serum of women diagnosed with preeclampsia. Notably, these increased concentrations were detectable as early as the second trimester, well before the clinical onset of symptoms.

This temporal profile positions miR-210 as a potential predictive marker for preeclampsia, particularly when combined with established clinical or biochemical indicators such as mean arterial pressure or the sFlt-1/PlGF ratio [38].

A meta-analysis encompassing more than 800 participants concluded that circulating miR-210 could distinguish between preeclamptic and normotensive pregnancies with a pooled sensitivity and specificity exceeding 80 percent [39].

The utility of miR-210 as a biomarker is further enhanced by its biological relevance to disease pathogenesis. Unlike markers that reflect secondary damage, miR-210 levels correlate directly with hypoxic stress, oxidative imbalance, and placental dysfunction. These mechanisms are not only central to the development of preeclampsia but are also present in its early stages, making miR-210 a mechanistically anchored candidate for risk assessment [40].

Technical advantages also support the clinical viability of miR-210. MicroRNAs are remarkably stable in biological fluids, resistant to RNase degradation, and can be reliably quantified using standardised protocols such as RT-qPCR. In addition, miR-210 remains detectable in small plasma volumes, allowing for convenient and minimally invasive sampling during routine prenatal visits [41].

Despite its promise, certain limitations must be addressed before routine clinical application. There is currently a lack of standardised cut-off values for miR-210 levels, and the variability between studies in methodology and patient populations may affect reproducibility. Moreover, comorbid conditions such as gestational diabetes or systemic inflammation may also influence circulating miRNA profiles, potentially confounding diagnostic interpretations [42,43].

### 1.4. Therapeutic Potential of Targeting miR-210

Given the central role of miR-210 in the molecular pathways underlying preeclampsia, its therapeutic targeting has garnered increasing interest. Unlike traditional treatments which primarily manage symptoms such as hypertension or proteinuria, interventions aimed at modulating miR-210 offer the possibility of addressing upstream molecular dysfunctions associated with placental pathology [44]. Experimental models have demonstrated that overexpression of miR-210 in trophoblast cells leads to impaired invasion, disrupted mitochondrial metabolism, and heightened oxidative stress. These effects contribute directly to poor placentation, a defining feature of preeclampsia.

Conversely, silencing miR-210 using antisense oligonucleotides or miRNA sponges has been shown to restore normal trophoblast behaviour in vitro, enhancing cell migration, mitochondrial function, and angiogenic signalling [45]. Such findings support the rationale for developing miR-210 inhibitors as a therapeutic strategy. Several approaches have been explored to suppress miR-210 activity, including locked nucleic acid (LNA) antisense inhibitors, antagomirs, and small-molecule modulators of upstream regulators such as HIF-1α.

Among these, LNA-modified oligonucleotides have shown particular promise due to their high binding affinity, stability in biological fluids, and resistance to enzymatic degradation. In animal models of placental insufficiency, intrauterine or systemic delivery of LNA-anti-miR-210 has led to improvements in placental morphology and function without overt toxicity [46].

However, translating these preclinical findings into clinical therapies presents several challenges. Targeted delivery to the placenta remains a significant hurdle, as systemic administration risks off-target effects and may impact miR-210 function in other hypoxia-sensitive tissues such as the heart or brain. Advances in nanocarrier technology, such as placental-specific exosomes or lipid nanoparticles, may offer a solution by enhancing tissue specificity and reducing systemic exposure [47].

Another consideration is the timing of therapeutic intervention. Since miR-210 expression is most dysregulated during early placentation, treatments would likely need to be administered in the first or early second trimester, a window that requires early and reliable diagnosis. This further underscores the importance of integrating miR-210-based diagnostics with therapeutic strategies [48,49].

## 2. Materials and Methods

In conducting this systematic review, we adhered closely to the PRISMA (Preferred Reporting Items for Systematic Reviews and Meta-Analyses) guidelines, which are widely acknowledged for promoting transparency and methodological rigour in evidence synthesis [50,51]. Although this review has not been registered, the principles of the PRISMA framework were rigorously applied to enhance reproducibility and clarity.

**Figure 1:**
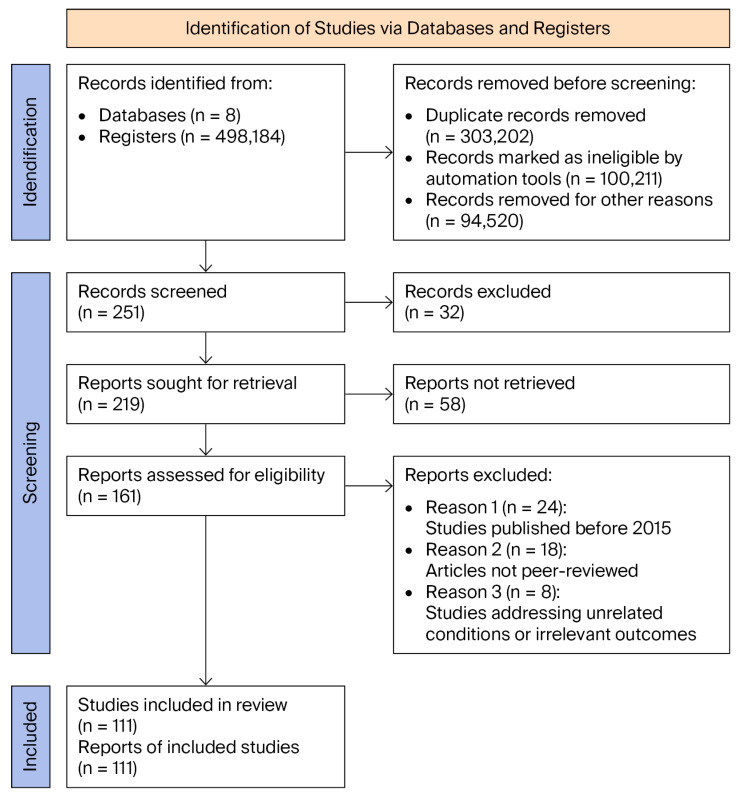
PRISMA flow diagram of articles related to role of microRNA-210 in pathogenesis and diagnosis of preeclampsia.

The comprehensive PRISMA flowchart shown in Figure 1 outlines the study selection process, detailing the inclusion and exclusion criteria. Additional clarification was provided to resolve inconsistencies observed between the initial and subsequent screening stages, following reviewer feedback regarding methodological transparency.

In this study, a systematic narrative analysis method was employed, combining the rigour of a systematic review with the flexibility of narrative synthesis. This methodological approach enabled the integrated aggregation and interpretation of information from multiple studies concerning the expression and function of microRNA miR-210 in the pathophysiological context of preeclampsia, offering a broad and coherent perspective on this emerging potential biomarker.

The synthesis of data from the specialist literature was conducted narratively, focusing on the molecular mechanisms involved, the variations in miR-210 expression in early- and late-onset forms of preeclampsia, and the clinical implications of these findings. The analysis went beyond merely presenting quantitative results, delving into existing knowledge gaps, potential diagnostic and therapeutic uses of miR-210, and future research directions.

This methodological choice was driven by the complex nature of preeclampsia, a multifactorial disorder involving placental hypoxia, vascular dysfunction, and epigenetic mechanisms. The aim was to integrate information from molecular biology, obstetrics, and predictive medicine to construct a robust explanatory framework.

The analysis was structured around three central axes:

- The function of miR-210 in response to placental hypoxia, through HIF-1α-regulated mechanisms and its effects on mitochondria and angiogenesis;
- Its potential role as a diagnostic and prognostic biomarker, assessed through its levels in plasma, serum, and placental tissue;
- The importance of an interdisciplinary approach in defining a molecular profile of preeclampsia, by correlating data from genetics, epigenetics, obstetrics, and placental pathology.

A fundamental question that was addressed was the extent to which miR-210 might contribute to better understanding, early detection, and potential therapeutic intervention in preeclampsia, by integrating it into predictive models. The findings highlight the need for collaboration among specialists from diverse fields—from molecular researchers to clinicians—to translate laboratory discoveries into tools applicable in clinical practice. Additionally, challenges related to standardising miR-210 measurement methods and validating its clinical utility were identified, along with future research perspectives that may support its integration into personalised medicine.

To address the objectives of this systematic review, an extensive search was conducted across eight recognised scientific databases (Wiley Online Library, MDPI, Springer, De Gruyter, Taylor & Francis, Nature, Frontiers, and Elsevier).

The search strategy was developed in accordance with the PRISMA guidelines, employing combinations of Boolean operators (AND, OR) to refine the results and ensure comprehensive coverage of the relevant literature concerning the role of microRNA-210 in preeclampsia.

Search terms included phrases such as *microRNA-210*, *preeclampsia*, *hypoxia*, *trophoblast invasion*, *mitochondrial dysfunction*, *biomarkers*, and *placental pathology*. These terms were combined into structured queries to maximise relevance, for instance, *microRNA-210 AND preeclampsia AND hypoxia OR trophoblast invasion*.

Results were filtered based on publication year (2015–2025) and the availability of full-text articles in English.

### 2.1. Eligibility Criteria for Study Selection

#### 2.1.1. Inclusion Criteria

- Selected articles needed to address preeclampsia from a molecular perspective, with a particular focus on the role of microRNA-210. Publications were included if they provided relevant insights into the following aspects:
  ○ Dysregulation of miR-210 expression in placental tissue or maternal circulation in the context of preeclampsia.
  ○ Molecular mechanisms influenced by miR-210, such as mitochondrial function, trophoblast invasion, angiogenesis, and oxidative stress.
  ○ The potential of miR-210 as a non-invasive diagnostic biomarker for preeclampsia.
  ○ Experimental therapeutic approaches targeting the inhibition of miR-210.
- Included studies comprised original research articles, systematic reviews, meta-analyses, and peer-reviewed publications from indexed journals, ensuring both scientific quality and relevance.

#### 2.1.2. Exclusion Criteria

The following categories of articles were excluded:

- Studies not adhering to PRISMA methodological standards, as compliance is essential for ensuring the rigour of the review process.
- Isolated case reports lacking generalisability or clear relevance to the molecular role of miR-210 in preeclampsia.
- Studies addressing unrelated pathologies or those in which miR-210 was not analysed in a placental context.

#### 2.1.3. Selection Process

All identified studies were assessed against clearly defined inclusion and exclusion criteria to ensure the relevance and quality of the final selection. The entire process was conducted in accordance with PRISMA guidelines, guaranteeing transparency and methodological consistency.

In the initial phase, selection was based on title and abstract screening, retaining only those studies that demonstrated general relevance to the investigation of miR-210 expression and function in the context of preeclampsia. The second phase involved a thorough full-text review, with inclusion limited to articles that clearly described molecular mechanisms influenced by miR-210 or highlighted its potential as a biomarker or therapeutic target. Accepted studies were subjected to a rigorous qualitative evaluation to confirm PRISMA compliance and alignment with the research objective. This methodology enabled a structured and stringent selection process, helping minimise the risk of excluding significant studies.

Nevertheless, it is important to acknowledge that the selection process may be affected by certain biases, including the omission of grey literature, the restriction to English-language publications, the exclusive use of eight major databases, and the limitation of the analysis period to 2015–2025. These methodological choices may result in the inadvertent exclusion of valuable perspectives from less visible sources or older studies.

### 2.2. Assessment of Risk of Bias and Robustness of Evidence

To enhance the transparency of the methodological process and ensure the credibility of the conclusions drawn in this narrative systematic review, a general assessment of both the risk of bias and the quality of the evidence was conducted during the full-text analysis phase. This approach was inspired by the Cochrane methodology for evaluating bias in randomised clinical trials, suitably adapted to the context of observational studies in the field of epigenetic and molecular research. The evaluation considered factors such as the transparency of the study design, the consistency of result reporting, and the appropriateness of the inclusion criteria applied. Given the narrative nature of the synthesis and the complexity of the subject matter, a detailed checklist was not applied to each individual study. Instead, an integrative assessment of the overall quality of the evidence was undertaken. Methodological limitations identified across the analysed works including potential sources of bias related to population selection, heterogeneity in experimental approaches, and the absence of a standardised protocol for quantifying miR-210 expression were explicitly recognised and discussed in the sections dedicated to result interpretation.

This evaluative strategy was deemed appropriate for balancing the breadth of the literature reviewed with the specific rigour required of a narrative review. It thereby contributed to a coherent and contextually grounded understanding of the implications of miR-210 in the pathogenesis of preeclampsia.

## 3. Results

### 3.1. Overview of Selected Studies

The PRISMA flowchart shown in Figure 1 illustrates the process of selecting articles related to the role of microRNA-210 in the pathogenesis and diagnosis of preeclampsia.

Each platform was queried using an approach tailored to its specific interface, and the snowballing technique was applied to include relevant articles cited in already selected studies.

Articles were retrieved from databases (*n* = 8) and scientific registries (*n* = 498,184). Prior to the screening stage, duplicates (*n* = 303,202), automatically flagged ineligible records (*n* = 100,211), and articles removed for other reasons (*n* = 94,520) were excluded.

Following this filtration, a total of 251 articles were included for screening, of which 32 were excluded. Full-text retrieval was attempted for the remaining 219 articles, though 58 could not be obtained.

A total of 161 articles were assessed for eligibility. Subsequently, those published before 2015 (*n* = 24), not peer-reviewed (*n* = 28), or dealing with conditions unrelated to the research topic or yielding irrelevant results (*n* = 9) were excluded.

A final total of 111 studies were included in the analysis.

### 3.2. Pathogenic Mechanisms Involved in Preeclampsia: The Role of miR-210

In the context of preeclampsia, molecular disturbances linked to the upregulation of microRNA-210 significantly contribute to the disruption of trophoblastic functions, angiogenesis, mitochondrial metabolism, and activation of the HIF-1α signalling pathway. Table 1 summarises the key pathogenic mechanisms associated with the overexpression of miR-210 in the placenta, along with their clinical implications.

Overall, miR-210 acts as a pathological regulator of placental homeostasis, and understanding this role offers promising directions for early diagnosis, risk stratification, and the development of targeted therapeutic interventions.

### 3.3. Diagnostic and Prognostic Value of miR-210 in Preeclampsia

To better understand the clinical potential of microRNA-210 in the context of preeclampsia, a comparative analysis of its use as a diagnostic biomarker versus a prognostic indicator is useful. Table 2 provides a detailed overview of the characteristics, advantages, limitations, and practical implications associated with both dimensions of miR-210’s applicability.

Overall, miR-210 holds considerable potential as a valuable diagnostic and prognostic tool in the management of preeclampsia. Continued research into its clinical validation, integration into multi-marker panels, and the definition of standardised protocols could play a decisive role in improving the monitoring of high-risk pregnancies and reducing maternal–foetal complications associated with this condition.

The comparison of these two applications of miR-210 highlights its dual role in obstetric practice. As a diagnostic biomarker, miR-210 offers a complementary method for confirming preeclampsia. Meanwhile, its use as a prognostic marker opens new avenues for preventive interventions and personalised monitoring. Integrating miR-210 into multi-marker panels and conducting broader clinical validation could position it as a key tool in modern maternal–foetal medicine.

The role of microRNA-210 in the pathogenesis of preeclampsia is highlighted through multiple interconnected molecular dysfunctions that impair the development and normal function of the placenta. The upregulation of miR-210 expression in placental tissue, consistently observed in both experimental and clinical studies, contributes to the emergence of a pathological cascade involving the trophoblast, vascular network, and cellular metabolism.

Trophoblastic invasion, a process essential for effective placentation, is compromised due to the suppression of key genes involved in extracellular matrix degradation and cellular motility. This results in shallow invasion and incomplete remodelling of the spiral arteries—central aspects in the pathophysiology of preeclampsia.

In addition to its impact on invasion, miR-210 impairs angiogenesis by inhibiting pro-angiogenic signalling, particularly through the downregulation of EFNA3. Consequently, vascular development is restricted, and trophoblast–endothelium interactions become inefficient, directly affecting uteroplacental blood flow.

At the mitochondrial level, miR-210 interferes with the respiratory chain, reducing ATP production and amplifying oxidative stress. This energy dysfunction is especially detrimental in trophoblasts, where metabolic demands are high during the early stages of pregnancy. Moreover, the accumulation of reactive oxygen species generates a pro-inflammatory environment and exacerbates placental tissue damage.

The HIF-1α signalling pathway, activated under hypoxic conditions, is directly involved in regulating miR-210 expression. Stabilisation of HIF-1α leads to the induction of miR-210, which, through its actions, further enhances hypoxia, creating a negative feedback loop. This vicious cycle between hypoxia, mitochondrial dysfunction, and persistent miR-210 expression amplifies placental impairment.

The clinical consequences of these mechanisms are reflected in the development of an immature and dysfunctional placenta, characterised by ischaemia, chronic inflammation, and the release of anti-angiogenic factors into the maternal circulation. These molecular disturbances are associated with hypertension, proteinuria, and the systemic complications observed in preeclampsia.

microRNA-210 has garnered significant interest in obstetric research due to its distinct expression profile in preeclampsia and its remarkable stability in maternal circulation. Its elevated plasma levels, associated with placental hypoxia and trophoblastic dysfunction, position it as a promising candidate for clinical use, both diagnostically and prognostically.

From a diagnostic perspective, miR-210 directly reflects the molecular disruptions occurring within the placenta, providing a valuable complement to classical markers such as blood pressure and proteinuria. Unlike these conventional indicators, which typically reflect late clinical manifestations, miR-210 offers insight into the subclinical stages of the disease, facilitating earlier and more specific identification of preeclampsia.

As a prognostic marker, its value lies in its ability to signal the presence of impending placental dysfunction, even before the onset of clinical symptoms. Studies have shown that elevated expression of miR-210 during the second trimester can predict the later development of preeclampsia, particularly in severe or early-onset forms. This characteristic makes it useful for risk stratification and the monitoring of susceptible pregnancies, allowing for the initiation of preventive measures or early interventions.

Its technical reliability is supported by high plasma stability, resistance to degradation, and compatibility with standard detection methods such as RT-qPCR. These features facilitate its integration into routine prenatal screening without requiring advanced laboratory infrastructure.

However, the clinical application of miR-210 remains limited by the lack of standardised thresholds and methodological variability across studies. Factors such as maternal comorbidities or systemic inflammation may influence circulating miRNA levels, necessitating further validation in diverse cohorts.

### 3.4. Therapeutic Perspectives in Targeting miR-210

The exploration of microRNA-210 as a therapeutic target in preeclampsia opens new avenues in maternal–foetal medicine, particularly through the possibility of intervening in the pathogenic mechanisms during the early stages of the disease. Unlike conventional treatments, which aim to control symptoms, strategies focused on inhibiting miR-210 offer the potential to correct placental molecular dysfunctions at their source [82,83].

Antisense oligonucleotide therapy, especially using locked nucleic acid (LNA)-modified inhibitors, has shown efficacy in preclinical models. Administration of LNA-anti-miR-210 has succeeded in restoring mitochondrial function, reducing oxidative stress, and enhancing trophoblastic invasion, key processes required for normal placentation [84,85]. Additionally, the use of “miRNA sponges” has demonstrated similar results in vitro, acting as decoys to neutralise excess miR-210 [86,87].

Recent approaches also focus on the indirect regulation of miR-210 by modulating the HIF-1α pathway, which drives its expression under hypoxic conditions. Pharmacological inhibitors of HIF-1α or its associated signalling pathways could reduce miR-210 expression, thereby mitigating its pathogenic impact on the placenta [88,89].

Nonetheless, the clinical application of these therapies faces considerable challenges. Targeted delivery to the placenta is difficult, as the systemic distribution of RNA-based molecules may affect other hypoxia-sensitive tissues, such as the brain or heart [90,91]. Proposed solutions include the use of nanocarriers, such as lipid nanoparticles or placenta-specific exosomes, which may enhance tissue specificity and reduce systemic toxicity [92,93].

Another major obstacle is determining the optimal timing for intervention. Since miR-210 is most active during the early stages of placentation, therapeutic interventions should ideally be implemented in the first or early second trimester [93,94]. This necessitates early and predictive identification of at-risk pregnancies, highlighting the importance of integrating miR-210-based diagnostics with therapeutic strategies.

Therefore, although therapies targeting miR-210 remain in the experimental phase, they represent a paradigm shift in the management of preeclampsia, from symptomatic treatment to precise molecular intervention. Their validation in rigorous clinical studies will be essential to transform this therapeutic potential into a clinical reality.

## 4. Discussion

The findings highlight that miR-210 functions as a central pathogenic regulator in preeclampsia, supporting the hypothesis that its increased expression is not merely a passive phenomenon, but an active contributor to the disruption of placental homeostasis. This observation aligns with previous studies that have consistently reported upregulations of miR-210 in the placental tissues and maternal blood of women with preeclampsia, directly associated with placental hypoxia and oxidative stress [94,95]. Thus, miR-210 is more than a passive biomarker; it actively influences target genes involved in trophoblastic invasion, angiogenesis, mitochondrial function, and HIF-1α regulation [96].

The observed reduction in trophoblastic invasion in our study is consistent with previously described molecular mechanisms: miR-210 suppresses the expression of genes such as THSD7A and MNT, resulting in shallow invasion and inadequate spiral artery remodelling. This dysfunction is consistent with the classical pathology of preeclampsia and with the literature demonstrating that miR-210 inhibition restores trophoblastic invasion in vitro [97].

Regarding angiogenesis, our results suggest that miR-210 suppresses EFNA3 signalling and reduces the efficiency of trophoblast–endothelium interactions. This mechanism is supported by studies showing that EFNA3 is a direct target of miR-210, and that angiogenesis inhibition is a significant factor in the pathogenesis of preeclampsia [98]. Additionally, the mitochondrial dysfunction identified in our analysis—marked by reduced ATP levels and increased ROS—reflects miR-210’s targeting of genes such as ISCU1/2 and SDHD, also reported in tumour cells and studied as hypoxia biomarkers [99]. The link between placental hypoxia, HIF-1α stabilisation, and the amplification of miR-210 expression is well-documented, supporting the hypothesis of a self-reinforcing feedback loop [100].

Collectively, the accumulated molecular effects—trophoblastic dysfunction, impaired angiogenesis, mitochondrial damage, and HIF-1α feedback—explain the clinical phenomena observed: hypoxia, chronic inflammation, and endothelial dysfunction [101]. The evidence supports therapies targeting miR-210, either directly through LNA antisense inhibitors or indirectly via HIF-1α modulation, in line with emerging therapeutic strategies [102].

The diagnostic and prognostic value of miR-210 offers multiple advantages. Diagnostically, its elevated plasma levels in response to placental hypoxia add a molecular dimension to classical markers such as blood pressure and proteinuria, and can aid in identifying preeclampsia in subclinical stages. The prognostic role of miR-210 is supported by studies demonstrating correlations between its expression in the second trimester and the later development of severe or early-onset preeclampsia [103,104]. Evaluations using combined markers such as MAP suggest that miR-210 could help distinguish between early- and late-onset forms of the disease.

Technically, RT-qPCR, microarrays, and RNA sequencing offer reliable detection methods, with miR-210 showing resistance to RNase degradation and compatibility with non-invasive prenatal analysis protocols [104]. Establishing universally accepted thresholds would facilitate its clinical integration, although a lack of methodological standardisation and the influence of comorbidities remain key limitations, necessitating prospective standardised studies [105].

The therapeutic perspectives are promising but fraught with challenges. Experimental use of LNA anti-miR-210 has restored mitochondrial function and trophoblastic invasion. “miRNA sponges” also present a viable alternative; however, achieving targeted delivery to the placenta remains difficult. Advanced delivery technologies, such as nanocarriers, placenta-specific exosomes, and HIF-1α inhibitors, may improve specificity, but require validation through rigorous clinical trials [106].

One major challenge that remains is the timing of intervention. As miR-210 is involved in early placentation, therapeutic strategies must be applied in the first or early second trimester, necessitating early screening protocols based on molecular detection [107]. Integration into a multi-marker panel (e.g., with sFlt-1/PlGF) could enhance diagnostic accuracy and provide a foundation for personalised therapies and preventative strategies.

In the context of recently published evidence, the results of this analysis confirm and extend previous observations regarding the functional and predictive role of miR-210 in preeclampsia. Systematic studies have reported elevated miR-210 expression in preeclamptic placentas and maternal blood, consistent with our findings [108,109]. Moreover, these studies have demonstrated that miR-210 negatively regulates trophoblastic invasion via targeting ISCU1/2, reinforcing the hypothesis of a coherent molecular mechanism linking non-coding RNA expression with the clinical manifestations of the disease. Recent meta-analyses have also reported sensitivities and specificities exceeding 80% for miR-210 as a predictive marker in high-risk pregnancies, supporting its positioning as a potentially valid clinical biomarker [110].

Nevertheless, current evidence carries some limitations. Many included studies were observational, with small sample sizes and methodological variations in miR-210 collection, processing, and quantification. The lack of standardisation between detection platforms (RT-qPCR vs. microarray vs. RNA sequencing) contributes to heterogeneity and limits the comparability of results. Furthermore, confounding factors such as gestational age, maternal comorbidities (e.g., diabetes, infections, systemic inflammation), or previous treatments are not always rigorously controlled, reducing the external validity of the conclusions.

The limitations of the review process include the absence of protocol registration in the PROSPERO database and the exclusion of grey literature, which may have contained relevant unpublished data. Furthermore, only English-language articles were included, potentially introducing a language selection bias. The search was limited to eight databases, and the review period was restricted to 2015–2025, meaning that some earlier but still scientifically valid studies may have been omitted.

The implications of these findings are multifaceted, impacting clinical practice, public health policy, and future research. Clinically, miR-210 could become part of a prenatal screening panel for high-risk pregnancies, particularly when combined with established markers such as sFlt-1 and PlGF. In practice, early detection of molecular disruptions associated with preeclampsia could enable proactive interventions, including intensive monitoring, low-dose aspirin supplementation, or optimal delivery planning. Screening of first trimester ultrasound markers, in addition with biochemical markers is very important and it should continue until the clinical onset [111,112], knowing that it is one of the most important factors in preterm birth, [113] according to our internal protocol [114].

From a public health perspective, the integration of biomarkers like miR-210 into standard screening protocols could reduce the incidence of maternal–foetal complications and help lower the costs associated with preterm birth or emergency interventions.

Future research directions should include longitudinal studies on large cohorts that are ethnically and socio-economically diverse, transnational validation of miR-210 expression thresholds, and exploration of its combination with other relevant microRNAs.

Therefore, this analysis provides robust evidence that miR-210 not only reflects but actively contributes to the pathogenesis of preeclampsia. Its clinical and translational validation could mark a pivotal step toward personalised medicine in high-risk pregnancies and the development of innovative epigenetic therapies for one of the most severe obstetric complications.

## 5. Conclusions

The role of microRNA-210 in the pathogenesis of preeclampsia is underscored by multiple interconnected molecular dysfunctions that impair the normal development and function of the placenta. The consistent upregulation of miR-210 expression in placental tissue, as observed in both experimental and clinical studies, contributes to the onset of a pathological cascade involving the trophoblast, vascular network, and cellular metabolism.

Trophoblastic invasion, a process essential for effective placentation, is compromised due to the suppression of key genes involved in extracellular matrix degradation and cell motility. This results in shallow invasion and incomplete remodelling of the spiral arteries—central features in the pathophysiology of preeclampsia.

Beyond its impact on invasion, miR-210 affects angiogenesis by inhibiting pro-angiogenic signalling, particularly through the downregulation of EFNA3. Consequently, vascular development is impaired, and trophoblast–endothelium interactions become inefficient, directly affecting uteroplacental blood flow.

At the mitochondrial level, miR-210 interferes with the function of the respiratory chain, reducing ATP production and amplifying oxidative stress. This energy dysfunction is particularly detrimental in trophoblasts, which have high metabolic demands during early pregnancy. In addition, the accumulation of reactive oxygen species generates a pro-inflammatory environment and exacerbates placental tissue damage.

The HIF-1α signalling pathway, activated under hypoxic conditions, is directly involved in regulating miR-210 expression. Stabilisation of HIF-1α leads to miR-210 induction, which, in turn, exacerbates hypoxia, creating a negative feedback loop. This vicious cycle of hypoxia, mitochondrial dysfunction, and persistent miR-210 expression amplifies placental damage.

The clinical consequences of these mechanisms manifest as an immature and dysfunctional placenta, characterised by ischaemia, chronic inflammation, and the release of anti-angiogenic factors into the maternal circulation. These molecular disruptions are associated with hypertension, proteinuria, and the systemic complications typical of preeclampsia.

microRNA-210 has garnered particular interest in obstetric research due to its distinct expression profile in preeclampsia and its remarkable stability in maternal circulation. Its elevated plasma levels, linked to placental hypoxia and trophoblastic dysfunction, position it as a promising candidate for clinical application, both diagnostically and prognostically.

From a diagnostic standpoint, miR-210 directly reflects the molecular disturbances occurring in the placenta, offering a valuable complement to classical markers such as blood pressure and proteinuria. Unlike these traditional indicators, which reflect late clinical manifestations, miR-210 provides insights into the subclinical stages of the disease, contributing to the earlier and more specific identification of preeclampsia.

As a prognostic marker, its value lies in its ability to signal imminent placental dysfunction even before clinical symptoms appear. Studies have shown that increased miR-210 expression in the second trimester can predict the subsequent development of preeclampsia, particularly in severe or early-onset forms. This makes it useful for the risk stratification and monitoring of at-risk pregnancies, allowing the initiation of preventive measures or early interventions.

Its technical reliability is supported by high plasma stability, resistance to degradation, and compatibility with standard detection methods such as RT-qPCR. These factors facilitate its integration into routine prenatal screening without the need for sophisticated laboratory infrastructure.

However, the clinical application of miR-210 remains limited by the absence of standardised thresholds and methodological variability between studies. Factors such as maternal comorbidities or systemic inflammation can influence circulating miRNA levels, necessitating further validation in diverse cohorts.

Overall, miR-210 holds considerable potential as a valuable diagnostic and prognostic tool in the management of preeclampsia. Continued research into its clinical validation and integration into multi-marker panels, as well as the development of standardised protocols, could make a decisive contribution to improving the monitoring of high-risk pregnancies and reducing maternal–foetal complications associated with this condition.

## Figures and Tables

**Table 1:** The role of miR-210 in placental molecular dysfunction in preeclampsia.

Aspect	Details	References
Primary Objective	miR-210 is a hypoxia-induced microRNA, upregulated in preeclampsia. Its primary role in placental molecular dysfunction lies in the inhibition of genes involved in mitochondrial metabolism, angiogenesis, and trophoblast invasion, thereby contributing to placental insufficiency and the pathogenesis of preeclampsia.	[52,53,54,55]
Trophoblast Dysfunction	miR-210 inhibits trophoblast invasion by suppressing the expression of genes essential for cellular motility (e.g., MNT, THSD7A), leading to shallow invasion and deficient placentation.	[56,57]
Impaired Angiogenesis	miR-210-mediated repression of EFNA3 reduces angiogenic signalling, limiting vascular development and spiral artery remodelling.	[58,59]
Mitochondrial Dysfunction	miR-210 suppresses key genes such as ISCU1/2 and SDHD, disrupting the electron transport chain and increasing oxidative stress.	[60,61]
HIF-1α Pathway Activation	Under hypoxic conditions, HIF-1α induces miR-210, which in turn exacerbates hypoxia by inhibiting mitochondrial function, forming a pathological self-perpetuating loop.	[62,63]
Cumulative Effects	Sustained miR-210 expression contributes to a pro-inflammatory placental environment, reduced uteroplacental perfusion, and systemic endothelial dysfunction.	[64,65]
Clinical Significance	Understanding these mechanisms offers insights for the development of biomarkers and targeted therapies, with potential for early diagnosis and prevention of preeclampsia.	[39,66]

**Table 2:** Diagnostic and prognostic value of miR-210 in preeclampsia.

Aspect	Diagnostic Biomarker	Prognostic Biomarker	References
Definition	A molecular indicator detectable in plasma or serum, reflecting the presence of preeclampsia.	An early marker indicating the risk of developing preeclampsia before clinical symptoms emerge.	[67,68]
Mechanism	Elevated levels of miR-210 in maternal circulation in response to placental hypoxia.	Overexpression of miR-210 correlates with early molecular disturbances in trophoblast function and angiogenesis.	[69,70]
Timing of Detection	Detectable during the second and third trimesters, at symptomatic stages.	May be identified as early as the second trimester, prior to clinical onset.	[71,72]
Stability	Remarkably stable in plasma, resistant to RNase degradation, ideal for non-invasive testing.	Stable under varied storage conditions, suitable for prenatal screening.	[73]
Detection Tools	RT-qPCR, microarray, RNA sequencing.	Standardised techniques compatible with routine testing.	[74,75]
Clinical Benefits	Complements traditional markers (blood pressure, proteinuria) by providing molecular insight.	Enables early intervention and monitoring of high-risk pregnancies.	[76,77]
Limitations	Absence of universally accepted reference thresholds, inter-study variability.	Circulating miRNA profiles may be influenced by comorbidities.	[78,79]
Practical Relevance	Useful in differential diagnosis and confirmation of preeclampsia.	Aids in risk stratification and the individualisation of prenatal care.	[80,81]

## Data Availability

The database used is from the review. Considering data protection regulation, we cannot provide access to the original database.

## References

[1] Kokori E., Aderinto N., Olatunji G., Komolafe R., Babalola E.A., Isarinade D.T., Omoworare O.T. (2024). Prevalence and Materno-Fetal Outcomes of Preeclampsia/Eclampsia among Pregnant Women in Nigeria: A Systematic Review and Meta-Analysis. Eur. J. Med. Res..

[2] Thakur A.S., Tayade S., Patel D., Gupta A., Batra N., Batra N. (2024). Unraveling the Predictive Power: Placenta Growth Factor and Pregnancy-Associated Plasma Protein A in Pre-Eclampsia. Cureus.

[3] Nobles C.J., Mendola P., Mumford S.L., Silver R.M., Kim K., Andriessen V.C., Schisterman E.F. (2020). Preconception Blood Pressure and Its Change into Early Pregnancy: Early Risk Factors for Preeclampsia and Gestational Hypertension. Hypertension.

[4] Dines V., Suvakov S., Kattah A., Vermunt J., Narang K., Jayachandran M., Garovic V.D. (2023). Preeclampsia and the Kidney: Pathophysiology and Clinical Implications. Compr. Physiol..

[5] Zhou C., Zou Q.Y., Jiang Y.Z., Zheng J. (2020). Role of Oxygen in Fetoplacental Endothelial Responses: Hypoxia, Physiological Normoxia, or Hyperoxia?. Am. J. Physiol. Cell Physiol..

[6] Putra I.W.A. (2022). Molecular Development of Placenta and its Relationship with Preeclampsia and Fetal Growth Restriction. Eur. J. Med. Health Sci..

[7] Das S.K. (2025). Insight into the Etiology of Preeclampsia. Indian J. Biochem. Biophys..

[8] Margioula-Siarkou G., Margioula-Siarkou C., Petousis S., Margaritis K., Vavoulidis E., Gullo G., Mavromatidis G. (2022). The Role of Endoglin and Its Soluble Form in Pathogenesis of Preeclampsia. Mol. Cell. Biochem..

[9] Stepan H., Hund M., Andraczek T. (2020). Combining Biomarkers to Predict Pregnancy Complications and Redefine Preeclampsia: The Angiogenic-Placental Syndrome. Hypertension.

[10] Deng F., Lei J., Qiu J., Zhao C., Wang X., Li M., Gao Q. (2024). DNA Methylation Landscape in Pregnancy-Induced Hypertension: Progress and Challenges. Reprod. Biol. Endocrinol..

[11] Frazier S., McBride M.W., Mulvana H., Graham D. (2020). From Animal Models to Patients: The Role of Placental microRNAs, miR-210, miR-126, and miR-148a/152 in Preeclampsia. Clin. Sci..

[12] Aderinto N., Olatunji G., Kokori E., Sanker V., Yusuf I.A., Adefusi T.O., Awuah W.A. (2024). miR-210 in Ischaemic Stroke: Biomarker Potential, Challenges and Future Perspectives. Eur. J. Med. Res..

[13] Jaszczuk I., Koczkodaj D., Kondracka A., Kwaśniewska A., Winkler I., Filip A. (2022). The Role of miRNA-210 in Pre-Eclampsia Development. Ann. Med..

[14] Hu X.Q., Zhang L. (2021). Hypoxia and Mitochondrial Dysfunction in Pregnancy Complications. Antioxidants.

[15] Mammdoh Y.M., Omar H., Mohamed O.A., Abbas A.M., El-Din L.T. (2023). Predictive Value of microRNA-210 in Preeclampsia. J. Curr. Med. Res. Pract..

[16] Zaccagnini G., Greco S., Longo M., Maimone B., Voellenkle C., Fuschi P., Martelli F. (2021). Hypoxia-Induced miR-210 Modulates the Inflammatory Response and Fibrosis upon Acute Ischemia. Cell Death Dis..

[17] Linna-Kuosmanen S., Tomas Bosch V., Moreau P.R., Bouvy-Liivrand M., Niskanen H., Kansanen E., Kaikkonen M.U. (2021). NRF2 Is a Key Regulator of Endothelial microRNA Expression under Proatherogenic Stimuli. Cardiovasc. Res..

[18] Jin W., Wang J., Liu C.P., Wang H.W., Xu R.M. (2020). Structural Basis for Pri-miRNA Recognition by Drosha. Mol. Cell.

[19] Vergani-Junior C.A., Tonon-da-Silva G., Inan M.D., Mori M.A. (2021). DICER: Structure, Function, and Regulation. Biophys. Rev..

[20] Cao G., Fan P., Ma R., Wang Q., He L., Niu H., Luo Q. (2023). miR-210 Regulates Lung Adenocarcinoma by Targeting HIF-1α. Heliyon.

[21] Silina M.V., Dzhalilova D.S., Makarova O.V. (2023). Role of microRNAs in Regulation of Cellular Response to Hypoxia. Biochemistry.

[22] Li B., Dasgupta C., Huang L., Meng X., Zhang L. (2020). miRNA-210 Induces Microglial Activation and Regulates Microglia-Mediated Neuroinflammation in Neonatal Hypoxic-Ischemic Encephalopathy. Cell. Mol. Immunol..

[23] Chen Y.M., He X.Z., Wang S.M., Xia Y. (2020). δ-Opioid Receptors, microRNAs, and Neuroinflammation in Cerebral Ischemia/Hypoxia. Front. Immunol..

[24] Ma Q., Dasgupta C., Shen G., Li Y., Zhang L. (2021). MicroRNA-210 Downregulates TET2 and Contributes to Inflammatory Response in Neonatal Hypoxic-Ischemic Brain Injury. J. Neuroinflamm..

[25] Kim H.J., Kim G., Lee J., Lee Y., Kim J.H. (2022). Secretome of Stem Cells: Roles of Extracellular Vesicles in Diseases, Stemness, Differentiation, and Reprogramming. Tissue Eng. Regen. Med..

[26] Deng M., Tong R., Zhang Z., Wang T., Liang C., Zhou X., Hou G. (2021). EFNA3 as a Predictor of Clinical Prognosis and Immune Checkpoint Therapy Efficacy in Patients with Lung Adenocarcinoma. Cancer Cell Int..

[27] Arenas G.A., Lorca R.A. (2024). Effects of Hypoxia on Uteroplacental and Fetoplacental Vascular Function during Pregnancy. Front. Physiol..

[28] Cordier A.G., Zerbib E., Favier A., Dabi Y., Daraï E. (2024). Value of Non-Coding RNA Expression in Biofluids to Identify Patients at Low Risk of Pathologies Associated with Pregnancy. Diagnostics.

[29] Eccher A., Girolami I., Lucenteforte E., Troncone G., Scarpa A., Pantanowitz L. (2021). Diagnostic Mesothelioma Biomarkers in Effusion Cytology. Cancer Cytopathol..

[30] Bian X., Liu J., Yang Q., Liu Y., Jia W., Zhang X., Wang Y.L. (2021). MicroRNA-210 Regulates Placental Adaptation to Maternal Hypoxic Stress during Pregnancy. Biol. Reprod..

[31] Yagel S., Cohen S.M., Goldman-Wohl D., Beharier O. (2023). Redefining Pre-Eclampsia as Type I or II: Implementing an Integrated Model of the Maternal–Cardiovascular–Placental–Fetal Array. Ultrasound Obstet. Gynecol..

[32] Hemmatzadeh M., Shomali N., Yousefzadeh Y., Mohammadi H., Ghasemzadeh A., Yousefi M. (2020). MicroRNAs: Small Molecules with a Large Impact on Pre-Eclampsia. J. Cell. Physiol..

[33] Rencelj A., Gvozdenovic N., Cemazar M. (2021). MitomiRs: Their Roles in Mitochondria and Importance in Cancer Cell Metabolism. Radiol. Oncol..

[34] Mondal I., Gupta N., Sharma V., Sarkar C., Mishra D.P., Kulshreshtha R. (2024). ALDH5A1/miR-210 Axis Plays a Key Role in Reprogramming Cellular Metabolism and Has a Significant Correlation with Glioblastoma Patient Survival. Cancer Cell Int..

[35] Liang L., Chen Y., Wu C., Cao Z., Xia L., Meng J., Wang Z. (2023). MicroRNAs: Key Regulators of the Trophoblast Function in Pregnancy Disorders. J. Assist. Reprod. Genet..

[36] Alexander T., Roman I., Elena V., Andrei G. (2020). Publication-Based Analysis of miR-210 Dependent Biomarkers of Pre-Eclampsia. Biol. Commun..

[37] Hu X.Q., Dasgupta C., Song R., Romero M., Wilson S.M., Zhang L. (2021). MicroRNA-210 Mediates Hypoxia-Induced Repression of Spontaneous Transient Outward Currents in Sheep Uterine Arteries during Gestation. Hypertension.

[38] Ghosh S., Thamotharan S., Fong J., Lei M.Y., Janzen C., Devaskar S.U. (2024). Circulating Extracellular Vesicular microRNA Signatures in Early Gestation Show an Association with Subsequent Clinical Features of Pre-Eclampsia. Sci. Rep..

[39] Meghana K.S., Yaliwal R.G., Kadakol G.S., Bidri S.R., Kori S., Kadakol Sr G.S., Bidri S. (2025). Evaluation of miRNA-210 as a Prognostic Biomarker for Pre-Eclampsia: A Case-Control Study. Cureus.

[40] Zhang J., Luo Z., Zheng Y., Duan M., Qiu Z., Huang C. (2024). CircRNA as an Achilles Heel of Cancer: Characterization, Biomarker and Therapeutic Modalities. J. Transl. Med..

[41] Serati A., Novielli C., Anelli G.M., Mandalari M., Parisi F., Cetin I., Mandò C. (2023). Characterization of Maternal Circulating microRNAs in Obese Pregnancies and Gestational Diabetes Mellitus. Antioxidants.

[42] Wang H., Luo C., Wu X., Zhang J., Xu Z., Liu Y., Xie J. (2021). Circular RNA hsa_circ_0081343 Promotes Trophoblast Cell Migration and Invasion and Inhibits Trophoblast Apoptosis by Regulating miR-210-5p/DLX3 Axis. Reprod. Biol. Endocrinol..

[43] Zou Z., Forbes K., Harris L.K., Heazell A.E. (2021). The Potential Role of the ESRRG Pathway in Placental Dysfunction. Reproduction.

[44] Fang Z., Xu J., Zhang B., Wang W., Liu J., Liang C., Shi S. (2020). The Promising Role of Noncoding RNAs in Cancer-Associated Fibroblasts: An Overview of Current Status and Future Perspectives. J. Hematol. Oncol..

[45] Estrada-Meza C., Torres-Copado A., Loreti González-Melgoza L., Ruiz-Manriquez L.M., De Donato M., Sharma A., Paul S. (2022). Recent Insights into the microRNA and Long Non-Coding RNA-Mediated Regulation of Stem Cell Populations. 3 Biotech.

[46] Sayres L., Flockton A.R., Ji S., Rey Diaz C., Gumina D.L., Su E.J. (2023). Angiogenic Function of Human Placental Endothelial Cells in Severe Fetal Growth Restriction Is Not Rescued by Individual Extracellular Matrix Proteins. Cells.

[47] Kieran N.W., Suresh R., Dorion M.F., MacDonald A., Blain M., Wen D., Healy L.M. (2022). MicroRNA-210 Regulates the Metabolic and Inflammatory Status of Primary Human Astrocytes. J. Neuroinflamm..

[48] Hromadnikova I., Kotlabova K., Krofta L. (2024). Abnormal microRNA expression profile at early stages of gestation in pregnancies destined to develop placenta previa. Front. Med..

[49] Karadzov Orlic N., Joksić I. (2025). Preeclampsia pathogenesis and prediction—Where are we now: The focus on the role of galectins and miRNAs. Hypertens. Pregnancy.

[50] Page M.J., McKenzie J.E., Bossuyt P.M., Boutron I., Hoffmann T.C., Mulrow C.D., Shamseer L., Tetzlaff J.M., Akl E.A., Brennan S.E. (2021). The PRISMA 2020 statement: An updated guideline for reporting systematic reviews. BMJ.

[51] Shamseer L., Moher D., Clarke M., Ghersi D., Liberati A., Petticrew M., Shekelle P., Stewart L.A., PRISMA-P Group (2015). Preferred reporting items for systematic review and meta-analysis protocols (PRISMA-P) 2015: Elaboration and explanation. BMJ.

[52] Gerede A., Stavros S., Danavasi M., Potiris A., Moustakli E., Machairiotis N., Zikopoulos A., Nikolettos K., Drakakis P., Nikolettos N. (2025). MicroRNAs in Preeclampsia: Bridging Diagnosis and Treatment. J. Clin. Med..

[53] Giannubilo S.R., Cecati M., Marzioni D., Ciavattini A. (2024). Circulating miRNAs and Preeclampsia: From Implantation to Epigenetics. Int. J. Mol. Sci..

[54] Pankiewicz K., Fijałkowska A., Issat T., Maciejewski T.M. (2021). Insight into the Key Points of Preeclampsia Pathophysiology: Uterine Artery Remodeling and the Role of MicroRNAs. Int. J. Mol. Sci..

[55] Meruvu S., Ding Z., Choudhury M. (2024). Mono-(2-ethylhexyl) phthalate induces trophoblast hypoxia and mitochondrial dysfunction through HIF-1α-miR-210-3p axis in HTR-8/SVneo cell line. Curr. Res. Toxicol..

[56] Jairajpuri D.S., Malalla Z.H., Sarray S., Mahmood N. (2021). Analysis of differential expression of hypoxia-inducible microRNA-210 gene targets in mild and severe preeclamptic patients. Non-Coding RNA Res..

[57] Bavelloni A., Ramazzotti G., Poli A., Piazzi M., Focaccia E., Blalock W., Faenza I. (2017). MiRNA-210: A current overview. Anticancer Res..

[58] Bakirtzi K., Law I.K.M., Xue X., Iliopoulos D., Shah Y.M., Pothoulakis C. (2016). Neurotensin promotes the development of colitis and intestinal angiogenesis via Hif-1α–miR-210 signaling. J. Immunol..

[59] Huang X. (2017). Regulation of the hypoxic response by non-coding RNAs. Tumor Hypoxia.

[60] Troise D., Infante B., Mercuri S., Netti G.S., Ranieri E., Gesualdo L., Pontrelli P. (2023). Hypoxic state of cells and immunosenescence: A focus on the role of the HIF signaling pathway. Biomedicines.

[61] Srivani G., Imran M., Merchant N., Mandala J.P., Nagaraju G.P. (2022). Role of succinate dehydrogenase in hepatocellular carcinoma. Theranostics and Precision Medicine for the Management of Hepatocellular Carcinoma.

[62] Goyal A., Afzal M., Goyal K., Ballal S., Sharma G.C., Kavitha V., Ali H. (2025). miR-210: A non-invasive biomarker for hypoxia-driven lung cancer diagnosis and therapy. Clin. Chim. Acta.

[63] Kornacki J., Olejniczak O., Sibiak R., Gutaj P., Wender-Ożegowska E. (2024). Pathophysiology of Pre-Eclampsia—Two Theories of the Development of the Disease. Int. J. Mol. Sci..

[64] Bardan C.R., Ioniță I., Iordache M., Călămar-Popovici D., Todorescu V., Popescu R., Bernad E.S. (2024). Epigenetic Biomarkers in Thrombophilia-Related Pregnancy Complications: Mechanisms, Diagnostic Potential, and Therapeutic Implications: A Narrative Review. Int. J. Mol. Sci..

[65] Erbilen E.A., Varol G.F., Sut N., Turker N.P., Sayin N.C. (2024). Over-Expression of MicroRNA-210 and MicroRNA-185-5p in Normotensive Late-Fetal Growth Restriction: Preliminary Cohort Study. Gynecol. Obstet. Reprod. Med..

[66] Bigagli E., Spataro E., Pasquini L., Cinci L., D’Ambrosio M., De Blasi C., Luceri C. (2025). Vaginal miR-210-3p as a potential biomarker for pregnancies complicated by early fetal growth restriction: A proof-of-concept case-control study. Placenta.

[67] Salimbayeva D., Kurmanova A., Nurmakova A., Smailov M., Kypshakbayeva Z. (2025). Placental Biomarkers of Preeclampsia: Systematic Review. Bratisl. Med. J..

[68] Biró O., Alasztics B., Molvarec A., Joó J., Nagy B., Rigó Jr J. (2017). Various levels of circulating exosomal total-miRNA and miR-210 hypoxamiR in different forms of pregnancy hypertension. Pregnancy Hypertens..

[69] Trongpisutsak A., Phupong V. (2021). Prediction of preeclampsia using a combination of serum micro RNA-210 and uterine artery Doppler ultrasound. Sci. Prog..

[70] Nikuei P., Davoodian N., Tahamtan I., Keshtkar A.A. (2016). Predictive value of miR-210 as a novel biomarker for pre-eclampsia: A systematic review protocol. BMJ Open.

[71] Youssef H.M.G., Marei E.S. (2019). Association of MicroRNA-210 and MicroRNA-155 with severity of preeclampsia. Pregnancy Hypertens..

[72] Szczerba E., Kozyra-Pydyś E., Zajkowska A., Pankiewicz K., Szewczyk G., Maciejewski T., Fijałkowska A. (2025). Exploring the Role of miRNA-101a in the Circulatory System’s Adaptive Mechanisms in Hypertensive Disorders of Pregnancy. Diagnostics.

[73] Oancea M., Mihu D., Braicu C., Isachesku E., Nati I.-D., Boitor-Borza D., Diculescu D.M., Strilciuc S., Pană A. (2025). MicroRNAs in Preeclampsia: An Overview of Biomarkers and Potential Therapeutic Targets. Int. J. Mol. Sci..

[74] Shan Y., Hou B., Wang J., Chen A., Liu S. (2024). Exploring the role of exosomal MicroRNAs as potential biomarkers in preeclampsia. Front. Immunol..

[75] Tian J., Liu Y., Hu M., Zheng Y., Xu P., Zhang L., Kilby M.D. (2020). Upregulated LncZBTB39 in pre-eclampsia and its effects on trophoblast invasion and migration via antagonizing the inhibition of miR-210 on THSD7A expression. Eur. J. Obstet. Gynecol. Reprod. Biol..

[76] Hromadnikova I., Kotlabova K., Hympanova L., Krofta L. (2015). Cardiovascular and cerebrovascular disease associated microRNAs are dysregulated in placental tissues affected with gestational hypertension, preeclampsia and intrauterine growth restriction. PLoS ONE.

[77] Cirkovic A., Stanisavljevic D., Milin-Lazovic J., Rajovic N., Pavlovic V., Milicevic O., Milic N. (2021). Preeclamptic women have disrupted placental microRNA expression at the time of preeclampsia diagnosis: Meta-analysis. Front. Bioeng. Biotechnol..

[78] Kochhar P., Dwarkanath P., Ravikumar G., Thomas A., Crasta J., Thomas T., Mukhopadhyay A. (2022). Placental expression of miR-21-5p, miR-210-3p and miR-141-3p: Relation to human fetoplacental growth. Eur. J. Clin. Nutr..

[79] Awamleh Z., Han V.K. (2020). Identification of miR-210-5p in human placentae from pregnancies complicated by preeclampsia and intrauterine growth restriction, and its potential role in the pregnancy complications. Pregnancy Hypertens..

[80] Munaut C., Tebache L., Blacher S., Noël A., Nisolle M., Chantraine F. (2016). Dysregulated circulating miRNAs in preeclampsia. Biomed. Rep..

[81] Wei N., Song H. (2022). Circ-0002814 participates in proliferation and migration through miR-210 and FUS/VEGF pathway of preeclampsia. J. Obstet. Gynaecol. Res..

[82] Mora-Palazuelos C., Villegas-Mercado C.E., Avendaño-Félix M., Lizárraga-Verdugo E., Romero-Quintana J.G., López-Gutiérrez J., Bermúdez M. (2023). The role of ncRNAs in the immune dysregulation of preeclampsia. Int. J. Mol. Sci..

[83] Popova A.K., Vashukova E.S., Illarionov R.A., Maltseva A.R., Pachuliia O.V., Postnikova T.B., Glotov A.S. (2024). Extracellular Vesicles as Biomarkers of Pregnancy Complications. Int. J. Mol. Sci..

[84] Fasanaro P., Romani S., Voellenkle C., Maimone B., Capogrossi M.C., Martelli F. (2012). ROD1 is a seedless target gene of hypoxia-induced miR-210. PLoS ONE.

[85] Meng X., Zhang P., Zhang L. (2020). Fetal hypoxia impacts on proliferation and differentiation of Sca-1+ cardiac progenitor cells and maturation of cardiomyocytes: A role of microRNA-210. Genes.

[86] Avendaño-Portugal C., Montaño-Samaniego M., Guttman-Bazbaz R., Bravo-Estupiñan D.M., Ibáñez-Hernández M. (2025). Therapeutic Applications of Poly-miRNAs and miRNA Sponges. Int. J. Mol. Sci..

[87] Pozniak T., Shcharbin D., Bryszewska M. (2022). Circulating microRNAs in Medicine. Int. J. Mol. Sci..

[88] Adu-Gyamfi E.A., Cheeran E.A., Salamah J., Enabulele D.B., Tahir A., Lee B.K. (2024). Long non-coding RNAs: A summary of their roles in placenta development and pathology. Biol. Reprod..

[89] Dai W., Guo R., Na X., Jiang S., Liang J., Guo C., Li D. (2024). Hypoxia and the endometrium: An indispensable role for HIF-1α as therapeutic strategies. Redox Biol..

[90] Beermann J., Piccoli M.T., Viereck J., Thum T. (2016). Non-coding RNAs in development and disease: Background, mechanisms, and therapeutic approaches. Physiol. Rev..

[91] Mouillet J.F., Chu T., Sadovsky Y. (2011). Expression patterns of placental microRNAs. Birth Defects Res. A Clin. Mol. Teratol..

[92] Lycoudi A., Mavreli D., Mavrou A., Papantoniou N., Kolialexi A. (2015). miRNAs in pregnancy-related complications. Expert Rev. Mol. Diagn..

[93] Grossini E., Surico D., Venkatesan S., Ola Pour M.M., Aquino C.I., Remorgida V. (2025). Extracellular Vesicles and Pregnancy-Related Hypertensive Disorders: A Descriptive Review on the Possible Implications “From Bench to Bedside”. Biology.

[94] Muralimanoharan S., Maloyan A., Mele J., Guo C., Myatt L.G., Myatt L. (2012). MIR-210 modulates mitochondrial respiration in placenta with preeclampsia. Placenta.

[95] Colleoni F., Padmanabhan N., Yung H.W., Watson E.D., Cetin I., Tissot van Patot M.C., Murray A.J. (2013). Suppression of mitochondrial electron transport chain function in the hypoxic human placenta: A role for miRNA-210 and protein synthesis inhibition. PLoS ONE.

[96] Morales-Prieto D.M., Favaro R.R., Markert U.R. (2020). Placental miRNAs in feto-maternal communication mediated by extracellular vesicles. Placenta.

[97] Inno R., Kikas T., Lillepea K., Laan M. (2021). Coordinated expressional landscape of the human placental miRNome and transcriptome. Front. Cell Dev. Biol..

[98] Harapan H., Andalas M. (2015). The role of microRNAs in the proliferation, differentiation, invasion, and apoptosis of trophoblasts during the occurrence of preeclampsia—A systematic review. Tzu Chi Med. J..

[99] Fu G., Brkić J., Hayder H., Peng C. (2013). MicroRNAs in human placental development and pregnancy complications. Int. J. Mol. Sci..

[100] Skalis G., Katsi V., Miliou A., Georgiopoulos G., Papazachou O., Vamvakou G., Makris T. (2019). MicroRNAs in preeclampsia. Microrna.

[101] Chan S.Y., Loscalzo J. (2010). MicroRNA-210: A unique and pleiotropic hypoxamir. Cell Cycle.

[102] Luo R., Wang Y., Xu P., Cao G., Zhao Y., Shao X., Wang Y.L. (2016). Hypoxia-inducible miR-210 contributes to preeclampsia via targeting thrombospondin type I domain containing 7A. Sci. Rep..

[103] Anton L., DeVine A., Polyak E., Olarerin-George A., Brown A.G., Falk M.J., Elovitz M.A. (2019). HIF-1α stabilization increases miR-210 eliciting first trimester extravillous trophoblast mitochondrial dysfunction. Front. Physiol..

[104] Krawczynski K., Mishima T., Huang X., Sadovsky Y. (2016). Intact feto-placental growth in microRNA-210 deficient mice. Placenta.

[105] Tang Q., Gui J., Wu X., Wu W. (2019). Downregulation of miR-424 in placenta is associated with severe preeclampsia. Pregnancy Hypertens..

[106] Jelena M., Sopić M., Joksić I., Zmrzljak U.P., Karadžov-Orlić N., Egić A., Spasojević-Kalimanovska V. (2020). Placenta-specific plasma miR518b is a potential biomarker for preeclampsia. Clin. Biochem..

[107] Koushki M., Atan N.A.D., Omidi-Ardali H., Tavirani M.R. (2018). Assessment of correlation between miR-210 expression and pre-eclampsia risk: A meta-analysis. Rep. Biochem. Mol. Biol..

[108] Awoyemi T., Jiang S., Rahbar M., Logentherian P., Collett G., Zhang W., Vatish M. (2024). MicroRNA analysis of medium/large placenta extracellular vesicles in normal and preeclampsia pregnancies. Front. Cardiovasc. Med..

[109] Gan L., Liu Z., Wei M., Chen Y., Yang X., Chen L., Xiao X. (2017). MiR-210 and miR-155 as potential diagnostic markers for pre-eclampsia pregnancies. Medicine.

[110] Nejad R.M.A., Saeidi K., Gharbi S., Salari Z., Saleh-Gohari N. (2019). Quantification of circulating miR-517c-3p and miR-210-3p levels in preeclampsia. Pregnancy Hypertens..

[111] Trăistaru V.A., Zamfirescu V., Burnei Rusu A., Bohîlțea R., Nastasia Ș., Vlădăreanu R. Ultrasound markers in early pregnancy predictive for preeclampsia. Proceedings of the 5th Romanian Congress of the Romanian Society of Ultrasound in Obstetrics and Gynecology.

[112] Mitran M., Velicu O., Comandașu D.-E., Vlădăreanu S. (2017). Diagnosis and prevention of preeclampsia–literature review. Ginecol. Ro.

[113] Yalcin S.E., Ozler M.R., Yalcin Y., Saglam E., Yilmaz E.S., Nerez N. (2025). Association of Early Pregnancy Inflammatory Indices with Preterm Birth and Perinatal Outcomes in Pregnancies with Pregestational Diabetes. J. Clin. Med..

[114] Russu M., Stanculescu R., Nastasia S., Paun M., Mubarak N., Marin J.A., Lachanas I. (2009). Pregnancy Outcomes Following Preconception, Early and Late Administration of Vaginal Micronized Progesterone for Recurrent Pregnancy Loss.

